# Advances in the study of gut microecology and mechanisms of hyperuricemia and gouty arthritis

**DOI:** 10.3389/fimmu.2025.1738716

**Published:** 2026-01-08

**Authors:** Youliang Zhang, Hengyu Zhang, Tianwen Miao, Xuetao Wang, Yanan Zuo, Renwei Zhang, Liangtong Zhang, Yuan Cheng, Dong Liu, Xin Chen, Longcan Li, Xingwen Xie, Ning Li

**Affiliations:** 1 Gansu University of Traditional Chinese Medicine, Lanzhou, China; 2 Sichuan Provincial Orthopedic Hospital, Chengdu, China; 3 Affiliated Hospital of Gansu University of Traditional Chinese Medicine, Lanzhou, China

**Keywords:** gouty arthritis, gut microbes, gut microbiota, intestinal barrier, novel therapeutic perspectives

## Abstract

Gouty arthritis is a metabolic disorder caused by purine metabolism dysregulation, characterized by monosodium urate crystal deposition in and around joints, triggering acute articular inflammation via NLRP3 inflammasome activation and IL-1β-mediated inflammatory cascades. While hyperuricemia represents a critical biochemical prerequisite for gouty arthritis development, elevated serum urate levels do not invariably lead to the disease. Mounting evidence suggests a significant relationship between gut microbiota and the pathogenesis of both gouty arthritis and hyperuricemia. The gut microbial ecosystem influences host health through metabolic and immune function modulation, performing essential roles in digestion, energy harvesting, and short-chain fatty acid production. Intestinal dysbiosis can damage epithelial integrity, compromise immune tolerance, and activate immune cells, thus contributing to disease onset and progression. Elucidating the complex interactions between gut microbiota and the mechanisms underlying gouty arthritis and hyperuricemia presents promising opportunities for developing novel preventative and therapeutic interventions. This review synthesizes recent advances in understanding the gut-joint axis and evaluates emerging therapeutic strategies including probiotics, dietary interventions, and fecal microbiota transplantation.

## 1 Introduction

Gouty arthritis (GA) is a metabolic disorder resulting from dysregulated purine metabolism and the consequent hyperuricemia, characterized by the deposition of monosodium urate crystals within and around joints, leading to tissue and organ damage (1). Clinically, it manifests with hallmark inflammatory signs, including redness, swelling, heat, and pain in the affected joints and surrounding soft tissues. While the initial presentation commonly involves the first metatarsophalangeal joint, larger joints can also be affected, potentially triggering systemic acute inflammatory responses (1). Hyperuricemia, defined as elevated serum uric acid (UA) levels exceeding the saturation threshold (approximately 6.8–7.0 mg/dL), leads to the formation and deposition of monosodium urate crystals within the joint space, thereby inciting gouty inflammation. Although hyperuricemia is a critical biochemical prerequisite for GA, not all individuals with elevated serum urate levels develop the condition. As one of the most prevalent inflammatory arthritides, GA affects approximately 2–4% of the global population, with a higher incidence in men over 40 years of age (2). This condition frequently co-occurs with comorbidities such as obesity, coronary artery disease, hypertension, metabolic syndrome, and diabetes.

The gut microbiota encompasses the composition, functional attributes, and metabolic products of the intestinal microbial community, as well as the integrity of intestinal physical and immunological barriers. As one of the most complex microbial ecosystems in the human body, the gut microbiota plays a pivotal role in modulating intestinal, metabolic, and immune homeostasis (3). Its core metabolic functions include facilitating the digestion of complex carbohydrates, such as polysaccharides and oligosaccharides (4). Through diverse mechanisms—including energy harvesting, endotoxemia, short-chain fatty acids (SCFAs) production, and choline and bile acid metabolism—the gut microbiota influences host health via the gut-joint axis (5, 6). The intestinal mucosal immune system orchestrates surveillance and defense through coordinated cellular action at inductive and effector sites. Increased bacterial density within intestinal crypts can activate crypt epithelial cells to eliminate foreign pathogens (7). When gut immunity becomes dysregulated, pathogenic bacteria may breach the intestinal barrier (8), triggering disease through the activation of Toll-like receptors and related pathways (9). Research has established strong associations between gut microbiota and numerous human diseases, including rheumatoid arthritis (10), GA, hyperuricemia, inflammatory bowel disease (11), systemic lupus erythematosus (12), and diabetes (13), and other autoimmune and metabolic disorders. Recent investigations have further elucidated the intricate mechanisms by which gut microbiota influences purine metabolism and uric acid production, emphasizing the importance of individual gut microbial signatures in disease development. Gut dysbiosis can increase intestinal epithelial permeability, disrupt immune tolerance, activate immune cells, and ultimately contribute to joint inflammation and bone destruction (10).

## 2 Association between gut microbiota and hyperuricemia and gouty arthritis

A close relationship exists between GA and the gut microbiota. As the largest biological interface in the human body (250–400 m²), the gastrointestinal tract harbors over 1014 microorganisms that maintain host health through diverse physiological mechanisms (14). The gastrointestinal tract serves as a major route for UA excretion, accounting for approximately 30-40% of total elimination (15). This substantial contribution becomes even more critical in patients with chronic kidney disease, where impaired renal excretion shifts the burden of urate clearance toward the gut. The recognition of this gut-mediated excretion pathway has prompted intensive investigation into the bidirectional relationship between gut microbiota and UA homeostasis. Evidence indicates that the gut microbiota plays a pivotal role in regulating UA homeostasis, influencing both UA production and elimination. High-throughput sequencing studies have revealed specific gut microbial signatures in patients with GA. Compared to healthy controls, the microbial community in individuals with GA exhibits significant alterations in gene richness and diversity, characterized by an increased abundance of *Bacteroides caccae* and *B. xylanisolvens*, alongside a decreased abundance of *Faecalibacterium prausnitzii* and *Bifidobacterium pseudocatenulatum* (16). Metagenomic analysis has further confirmed an enrichment of *Prevotella*, *Fusobacterium*, and *Bacteroides* in GA patients, accompanied by a reduction in *Enterobacteriaceae* and butyrate-producing bacteria (17). In a rat model of hyperuricemic nephropathy, 16S rRNA analysis demonstrated a significant increase in opportunistic pathogens such as *Flavobacterium* and *Myroides*, coupled with a notable decrease in beneficial SCFA-producing bacteria such as *Blautia* and *Roseburia* (18). Recent investigations have identified an enrichment of *Phascolarctobacterium* and *Bacteroides* in individuals with GA, forming a characteristic “core microbiota” comprising three *Bacteroides* species (19). The specific roles and mechanisms of these and other key microbial taxa are systematically summarized in **Table 1**, which provides a comprehensive overview of their abundance changes in gout, main functions, and mechanistic contributions to disease pathogenesis.

**Table 1:** Key gut microbial taxa and their roles in hyperuricemia and gout pathogenesis.

Microbial taxa	Abundance	Main functions	Mechanisms	Refs
*Bacteroides caccae*	Increased	Amino acid metabolism	Enhanced BCAA catabolism; promotes XOD activity	(16)
*Prevotella* spp.	Increased	Pro-inflammatory	LPS production; TLR4/NF-κB activation	(17)
*Alistipes indistinctus*	Decreased	UA excretion	Hippuric acid production; ABCG2 upregulation	(24)
*Faecalibacterium prausnitzii*	Decreased	SCFA production	Butyrate producer; HDAC inhibition; NF-κB suppression	(16)
*Bifidobacterium* spp.	Decreased	Barrier protection	SCFA production; pathogen inhibition; Th17/Treg balance	(31, 32)
*Lactobacillus* spp.	Variable	UA degradation	Uricase production; XOD inhibition; purine absorption reduction	(22, 33)
*Escherichia coli*	Increased	UA production	Xanthine dehydrogenase secretion converts purines to UA	(20, 21)
*Akkermansia muciniphila*	Variable	UA transporter regulation	Modulates ABCG2, URAT1, GLUT9 expression	(34)
*Butyrate-producers (collective)*	↓ 40-60%	SCFA production	Collective depletion leads to SCFA deficiency and barrier dysfunction	(35)
*Purine-degrading bacteria*	Decreased	UA degradation	Harbor gene clusters for purine catabolism; compensate for uricase loss	(27, 28)

Intestinal microorganisms participate in the intricate regulation of purine and UA metabolism through multiple pathways. *Escherichia coli* and *Proteus* can secrete xanthine dehydrogenase, converting purines to UA (20, 21); conversely, *Lactobacillus* can lower serum UA levels by inhibiting intestinal purine absorption (22); and *Pseudomonas* can synthesize uricase, promoting UA catabolism (23). *Alistipes indistinctus* is significantly less abundant in patients with hyperuricemia. Experimental evidence demonstrates that supplementation with *A. indistinctus* can ameliorate hyperuricemia by enhancing intestinal UA excretion mediated by the *ATP-binding cassette transporter G2* (24). *A. indistinctus* can promote the production of hippuric acid, which facilitates UA excretion through a dual mechanism: by activating peroxisome proliferator-activated receptor γ to upregulate *ATP-binding cassette transporter G2* transcription, and by binding to PDZ domain-containing protein 1 to enhance *ATP-binding cassette transporter G2* trafficking to the brush border membrane, thereby augmenting its functional activity (24). Furthermore, studies have highlighted the significance of purine-degrading bacteria and their conserved gene clusters in UA elimination. These gene clusters encode enzymes crucial for the purine catabolic pathway. Various intestinal microorganisms secrete UA transporters, directly regulating UA excretion (25). The importance of purine-degrading bacteria and their conserved gene clusters in UA elimination is further emphasized by their encoded enzymes, which play a vital role in the purine catabolic pathway, underscoring the key contribution of gut microbes to UA homeostasis. Studies have identified diverse purine-degrading bacteria within the human gut microbiota, belonging to phyla such as Actinobacteria, Firmicutes, Clostridia, and Proteobacteria, capable of metabolizing UA into xanthine or SCFAs (26). These strains harbor a highly conserved gene cluster encoding key enzymes for UA degradation, which plays a central role in the purine catabolic pathway (27). In uricase-deficient mouse models, gut microbiota depletion via antibiotics significantly exacerbated hyperuricemia, with serum UA levels increasing by approximately 40-50%. Conversely, colonization with UA-degrading bacteria harboring conserved purine catabolic gene clusters (*ygeX, ygeY, ygeW, ygfK, ssnA*) effectively reduced UA levels by 30-35%, providing compelling experimental evidence for the critical compensatory role of these bacteria in maintaining UA homeostasis (28). Recent machine learning approaches have further validated the diagnostic potential of gut microbiota signatures. A predictive model incorporating the relative abundance of nine bacterial genera (including *Collinsella, Faecalibacterium*, and *Escherichia-Shigella*) achieved an area under the curve exceeding 0.85 for hyperuricemia prediction, outperforming traditional serum UA measurements alone (29). Notably, alpha-diversity measured by Shannon index was significantly lower in hyperuricemia groups compared to healthy controls, suggesting that microbial diversity loss itself may serve as an early indicator of metabolic dysregulation. Mendelian randomization studies have begun to establish causal relationships, confirming that Escherichia-Shigella is a risk factor for hyperuricemia, while Lachnospiraceae species and Family XIII reduce hyperuricemia risk (30).

## 3 Association between gut microbiota dysbiosis and the pathogenesis of hyperuricemia and gouty arthritis

The gut microbiota serves as a crucial regulator of human metabolic and immune system functions (36, 37). Contemporary research demonstrates that gut microbial influence extends significantly beyond the intestinal environment (38). Evidence from both animal models and clinical investigations has established a strong correlation between gut microbiota alterations and the development of hyperuricemia and GA. These findings suggest that gut microbial composition could function as a valuable biomarker for monitoring GA onset, disease progression, and clinical outcomes (39–41).

### 3.1 Mechanisms by which gut microbes and metabolite disorders cause hyperuricemia and gouty arthritis

**Figure 1:**
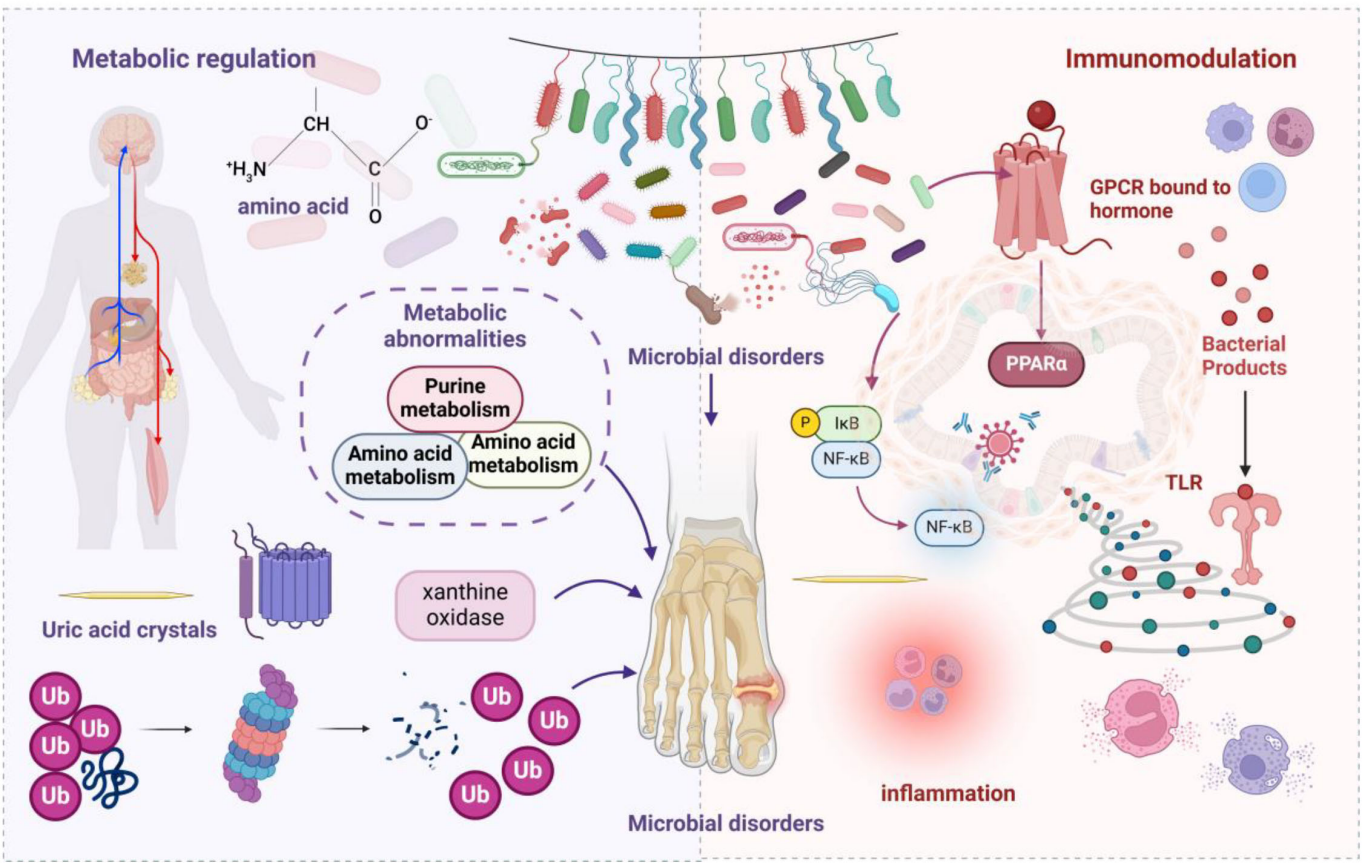
schematically illustrates hyperuricemia and GA pathogenesis driven by intestinal microbiota dysbiosis. The gut microbiota and their metabolic products exert a pivotal role in gout pathogenesis. The gut microbiome orchestrates gout-relevant metabolic processes via multiple pathways. Microbial dysbiosis can precipitate aberrant uric acid metabolism and immune system perturbations. Alterations in microbial community composition influence uric acid production, degradation, and excretion. In terms of immunomodulation, gut microbiota dysbiosis incites aberrant activation of innate immune cells, augmenting pro-inflammatory mediator expression, such as interleukin-12 and interleukin-23, while concurrently suppressing anti-inflammatory cytokine production, like interleukin-10 and transforming growth factor β, thereby impacting GA pathogenesis. Declines in microbial metabolite levels, such as SCFAs, induce immune regulatory dysfunction, fostering inflammatory cytokine release. This metabolic-immune network imbalance represents a crucial mechanism in gout development, providing potential interventional targets for disease prevention and therapy.

The gut microbiota and its metabolites play a significant role in the pathogenesis of hyperuricemia and GA. Studies have shown that the gut microbiota contributes to the metabolism of approximately one-third of systemic UA by transporting it from the blood to the intestinal lumen via secreted UA transporters (42). In a uricase-deficient mouse model, dysregulation of amino acid metabolism, impaired intestinal barrier function, and altered expression of solute carrier families collectively contribute to elevated serum UA levels and CD4+ Th17-mediated inflammatory responses (43). **Figure 1** schematically illustrates the mechanisms by which gut microbial dysbiosis contributes to hyperuricemia and GA.

#### 3.1.1 Metabolic regulation

The gut microbiota can modulate metabolic processes relevant to hyperuricemia and GA through multiple pathways. Metagenomic studies have revealed two distinct gut microbiota profiles in individuals with GA: the high-GA group, characterized by a predominance of *Bacteroides*, exhibits increased synthesis of alanine and branched-chain amino acid catabolic products (39); conversely, the low-GA group, dominated by *Faecalibacterium*, shows elevated production of butyrate, sulfur-containing amino acids, and their metabolite hydrogen sulfide (44). This compositional difference directly influences the metabolic landscape. Within the UA metabolism regulatory network, gut microbiota-driven metabolic disturbances in patients with hyperuricemia and GA lead to elevated amino acid levels, resulting in aberrant purine metabolism. This process is accompanied by enhanced xanthine oxidase (XOD) activity, which catalyzes the oxidation of hypoxanthine and xanthine to UA. Concurrently, lipopolysaccharide produced by Gram-negative bacteria can further amplify XOD synthesis and activity (45). A diverse array of gut microbes participates in UA degradation. Various probiotics reduce UA levels through distinct mechanisms: *Bifidobacterium* and *Lactobacillus* can inhibit XOD activity and encode UA-degrading enzymes; *Lactobacillus* can diminish intestinal purine absorption (22); and *Alistipes indistinctus* promotes UA excretion via the production of hippuric acid (24). These findings provide a scientific rationale for microbial-targeted therapies for hyperuricemia and its related conditions. *Bacillus pasteurii*, *Proteus mirabilis*, and *Escherichia coli* can produce uricase, converting UA into allantoin and urea (46). *Lactobacillus* OL-5 and *Lactobacillus plantarum* (*Mut-7, Dad-13*) exhibit high intracellular uricase activity (33). *Solute carrier family 2 member 9* and *ATP-binding cassette subfamily G member 2 (ABCG2)* are key proteins for UA transport (47, 48). As detailed in Section 3.1.3, SCFAs produced by the gut microbiota can enhance the expression of these transporters, thereby increasing UA excretion. However, in patients with hyperuricemia and GA, the production of SCFAs by the gut microbiota is diminished, leading to a reduction in the expression of UA transporters, hydrolases, and uricase in intestinal epithelial cells, ultimately impairing effective UA clearance (18, 49). This multifaceted metabolic regulatory network underscores the central role of the gut microbiota in the pathogenesis of hyperuricemia and GA.

#### 3.1.2 Immune regulation

The gut microbiota serves as a crucial mediator in immune regulation associated with hyperuricemia and GA through orchestrated activation of innate and adaptive immune responses. The following describes the key immune signaling pathways linking gut dysbiosis to gouty inflammation. The TLR4/NF-κB signaling pathway serves as the critical “priming signal” for inflammasome activation in gout pathogenesis. When intestinal barrier dysfunction occurs due to gut dysbiosis, increased translocation of lipopolysaccharide from Gram-negative bacteria into systemic circulation activates TLR4 on macrophages and monocytes (50). lipopolysaccharide binds to the TLR4-MD2 complex, triggering recruitment of adaptor proteins MyD88 and TRIF, which activate intracellular kinase cascades leading to phosphorylation and degradation of IκB. Released NF-κB translocates to the nucleus and induces transcription of NLRP3 inflammasome components (NLRP3, ASC, pro-caspase-1), pro-inflammatory cytokine precursors (pro-IL-1β, pro-IL-18), and other pro-inflammatory mediators (TNF-α, IL-6, COX-2) (50). This TLR4/NF-κB-mediated priming is essential but insufficient for IL-1β secretion; a second activation signal is required. The NLRP3 inflammasome represents the central molecular platform linking gut dysbiosis to gouty inflammation. This multiprotein complex consists of NLRP3, ASC, and pro-caspase-1. MSU crystals trigger inflammasome assembly through multiple mechanisms: potassium efflux, lysosomal rupture, mitochondrial dysfunction, and reactive oxygen species production (51). Once activated, NLRP3 oligomerizes and recruits ASC to form specks that concentrate pro-caspase-1, leading to its activation. Active caspase-1 cleaves pro-IL-1β to mature IL-1β and induces pyroptosis through gasdermin D cleavage, releasing inflammatory cytokines (52). Mature IL-1β serves as the master regulator for amplification of MSU crystal-induced inflammation. IL-1β binds to IL-1 receptor type 1, recruiting IL-1R accessory protein to form a signaling complex that activates MyD88-dependent pathways, including NF-κB, MAPK (p38, ERK, JNK), and PI3K/AKT cascades (50). This results in production of additional pro-inflammatory mediators (TNF-α, IL-6, chemokines) and creates an amplification loop. IL-1β exerts pleiotropic effects on multiple cell types: inducing vasodilation and increased vascular permeability in endothelial cells, promoting massive neutrophil recruitment via chemokines, enhancing macrophage activation, stimulating synovial fibroblasts to produce matrix metalloproteinases, and activating osteoclasts leading to bone erosion (52). Gut microbiota dysbiosis disrupts the balance between pro-inflammatory Th17 cells and anti-inflammatory regulatory T cells. Decreased production of SCFAs by depleted commensal bacteria reduces *G protein-coupled receptor 43* and *GPR109a* signaling, impairing Foxp3+ Treg differentiation in gut-associated lymphoid tissue (53, 54). Simultaneously, increased LPS and pathogenic bacterial antigens activate dendritic cells to produce IL-6, IL-23, and IL-1β, promoting Th17 differentiation. Th17 cells produce IL-17A, which synergizes with IL-1β to amplify neutrophil recruitment and induce synovial fibroblast activation (55). In hyperuricemia and GA patients, decreased SCFA levels result in Treg depletion and PPARγ downregulation, exacerbating immune dysregulation with Th17/Treg ratios increasing from ~0.5-1.0 in healthy individuals to >2.0 in gout patients (39, 56).

#### 3.1.3 The central role of short-chain fatty acids in gut-gout pathogenesis

SCFAs, predominantly butyrate, acetate, and propionate, serve as critical mediators linking gut microbiota composition to gout pathogenesis through three major mechanisms. SCFAs produced by commensal bacteria enhance the expression of intestinal UA transporters, particularly *ABCG2* and *GLUT9/SLC2A9* (47, 48). Butyrate directly upregulates *ABCG2* transcription through histone deacetylase inhibition, thereby increasing UA secretion from intestinal epithelial cells into the gut lumen (35). In hyperuricemia and gout patients, depletion of SCFA-producing bacteria leads to reduced *ABCG2* and uricase expression in intestinal epithelial cells, impairing effective UA clearance (18, 49). SCFAs modulate immune responses through multiple pathways. Butyrate suppresses NF-κB activation and enhances peroxisome proliferator-activated receptor γ expression in intestinal epithelial cells, resulting in decreased pro-inflammatory cytokine production (35). *In vitro* studies demonstrate that butyrate inhibits HDAC activity, reducing MSU crystal-induced production of IL-1β and IL-18 (52). Acetate mediates neutrophil caspase-dependent apoptosis through *G protein-coupled receptor 43*, inhibiting *NLRP3* inflammasome activation and promoting inflammation resolution (52). Propionate enhances regulatory T cell differentiation via *G protein-coupled receptor 43* signaling, maintaining immune homeostasis (54). In hyperuricemia and gout, decreased SCFA levels result in Treg depletion and peroxisome proliferator-activated receptor downregulation, exacerbating immune dysregulation (39, 56). Butyrate serves as the primary energy source for colonocytes, supporting ATP production necessary for tight junction protein maintenance (35). SCFAs stabilize the epithelial mucosal barrier by promoting repair of intestinal epithelial cells and enhancing expression of tight junction proteins ZO-1, occludin, and claudin-1 (51). Acetate provides additional energy support for intestinal epithelial cells and facilitates UA transport (57, 58). However, in hyperuricemia and gout patients, significantly reduced levels of *Bifidobacterium* and other SCFA-producing bacteria lead to weakened barrier protection and accelerated disease progression (31, 32). The central role of SCFAs suggests that therapeutic strategies aimed at restoring SCFA production through probiotic supplementation, prebiotic administration, or dietary fiber intake may effectively target multiple pathological mechanisms simultaneously. This multimodal action profile makes SCFA modulation an attractive therapeutic approach for gout management.

### 3.2 Mechanism of gut barrier dysfunction in hyperuricemia and gouty arthritis

**Figure 2:**
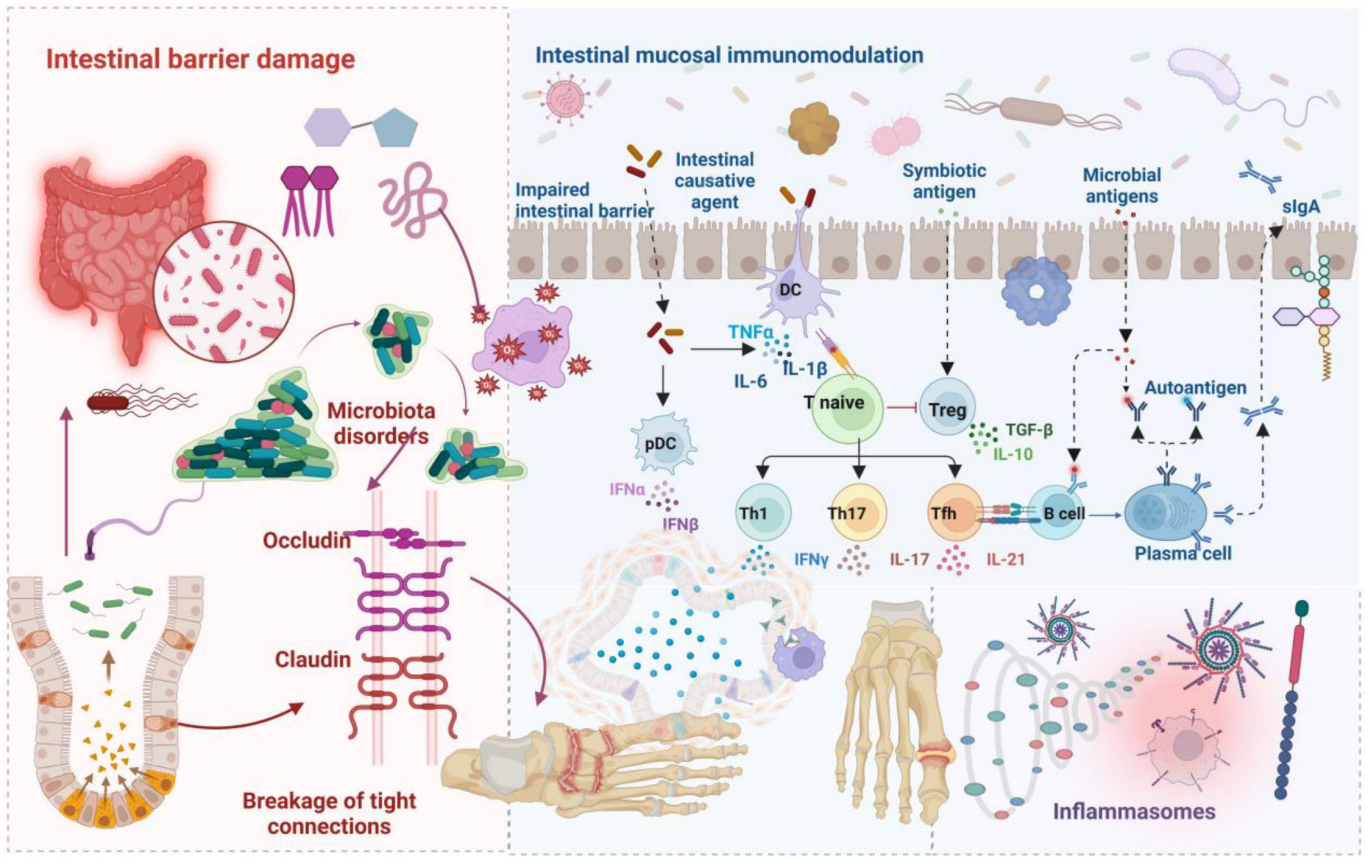
schematically illustrates the intestinal barrier’s influence on hyperuricemia and GA. The intestinal barrier impacts GA pathogenesis via multiple mechanisms. Elevated intestinal permeability, resulting from diminished epithelial tight junction protein expression, exhibits a positive correlation with UA levels; the intestinal mucosal immune system constitutes a complex network integrating innate, adaptive, and IgA immune responses. Epithelial and dendritic cells recognize antigens via pattern recognition receptors, presenting them to T and B lymphocytes in lymphoid tissues to initiate adaptive immune responses and activate signaling pathways to induce cytokine and antimicrobial peptide production, triggering acute inflammatory responses to eradicate pathogens. B lymphocytes differentiate into IgA plasma cells, producing secretory IgA transported to the intestinal lumen to neutralize antigens and pathogens, representing the principal mucosal immunity defense. Intestinal mucosal barrier disruption can incite various inflammations, thus promoting GA progression.

The intestinal barrier comprises a sophisticated multi-layered defense system consisting primarily of gut microbiota, the mucus layer, epithelial cell monolayer, and immune cells within the lamina propria (59). These components work synergistically to maintain intestinal microecological homeostasis through complementary mechanisms. The mucus layer and epithelial cell monolayer constitute a physical barrier that effectively prevents bacterial adhesion and translocation (5, 6). Concurrently, the lamina propria and submucosa tissues mediate immune responses that defend against invasion by both commensal and pathogenic microorganisms (60). Within the epithelial barrier, intercellular tight junction proteins play a critical role in maintaining barrier integrity by regulating transepithelial permeability and cell mechanical connections (61, 62). As a core component of the intestinal barrier, epithelial cells are continuously renewed by stem cells located at the base of the crypts, providing not only a physical barrier but also preventing commensal bacteria from invading host tissues through biochemical mechanisms (7), thereby influencing the development of various diseases, including hyperuricemia and GA. **Figure 2** schematically illustrates the mechanisms by which gut barrier dysfunction contributes to hyperuricemia and GA. The distinct contributions of each barrier type and their dysfunction patterns in gout are systematically presented in **Table 2**.

**Table 2:** Intestinal barrier components and their dysfunction in hyperuricemia and gout.

Barrier type	Components	Dysfunction in gout	Key molecules	Refs
Mucus Layer	Mucin glycoproteins; Glycocalyx	Reduced mucin production; thinning of mucus layer; decreased A. muciniphila	MUC2, MUC3, MUC4	(34, 63)
Tight Junction Complex	Occludin, Claudins, ZO-1	↓ Occludin (30-50%); ↓ Claudin-1 (40-60%); ↓ ZO-1 (35-55%); ↑ Claudin-2 (2-3×); ↑ Zonulin (2-3×)	Occludin, ZO-1, Claudin-1, Claudin-2, Zonulin	(57, 58, 64–66)
Innate Immunity	Macrophages, DCs, Neutrophils, IECs	↑ TLR4; M1 polarization; NLRP3 activation; ↑ IL-1β, TNF-α; excessive neutrophil infiltration	TLR4, NLRP3, IL-1β, IL-18, TNF-α	(50–52, 67)
Adaptive Immunity	CD4+ T cells, CD8+ T cells, B cells	Th1 polarization; Th17 expansion (Th17/Treg >2.0); Treg depletion (↓ 40-60%); altered IgA	Th17, Tregs, IL-17A, Foxp3, IgA	(53, 54, 68)
Antimicrobial Peptides	Defensins, Cathelicidins, RegIIIγ	Reduced β-defensins; decreased RegIIIγ; loss of bacterial spatial segregation	α-defensins, β-defensins, LL-37, RegIIIγ	(7, 59, 63)
SCFAs	Butyrate, Acetate, Propionate	SCFA depletion (40-60%): ↓ Butyrate (20-30→8–15 mM); ↓ Propionate; ↓ Acetate (see Section 3.1.3)	Butyrate, Acetate, Propionate, GPR43, HDAC	(18, 35, 49, 53, 54)

#### 3.2.1 Impact of the intestinal physical barrier on hyperuricemia and gouty arthritis

The intestinal physical barrier comprises the epithelial layer, the underlying lamina propria, and the muscularis mucosa, with the surface mucus layer and intercellular tight junctions forming the primary defensive barrier (69, 70). In the pathogenesis of hyperuricemia and GA, aberrant expression of tight junction proteins leads to barrier dysfunction (**Table 2**). Zonulin, a physiological modulator of tight junction permeability, is significantly upregulated in gout patients (64, 65). Elevated zonulin disrupts barrier integrity through a dual mechanism: proteolytic degradation of ZO-1 and occludin, and transcriptional upregulation of pore-forming claudins (claudin-2 and claudin-15), which form paracellular channels permeable to small ions and water (66). This altered tight junction protein expression profile results in markedly increased intestinal permeability, as evidenced by elevated serum zonulin levels correlating positively with serum UA concentrations (57, 58). A complex interplay exists between intestinal physical barrier dysfunction and gut microbiota dysbiosis. Barrier damage contributes to gut microbiota dysbiosis, and the imbalanced microbiota produces metabolites such as hydrogen sulfide, reactive oxygen species, and reactive nitrogen species, which directly damage the structure and function of intestinal epithelial cells (71). This exacerbated damage further increases intestinal permeability, triggering bacterial translocation and establishing a vicious cycle that promotes inflammation and GA development. Conversely, microbial metabolites play a protective role in maintaining intestinal barrier function. As described in Section 3.1.3, butyrate stabilizes the epithelial mucosal barrier by promoting the repair of intestinal epithelial cells, while acetate provides energy support for intestinal epithelial cells and facilitates UA transport (57). *Bifidobacterium* improves mucosal barrier function by inhibiting the proliferation of harmful bacteria (32). However, in patients with hyperuricemia and GA, the levels of *Bifidobacterium* and SCFAs are significantly reduced, leading to weakened barrier protection and ultimately accelerating GA progression (32).

#### 3.2.2 Impact of the intestinal immune barrier on hyperuricemia and gouty arthritis

The intestinal immune barrier comprises the lamina propria and its resident immune cells, consisting primarily of intestinal epithelial cells, diverse immune cell populations within the lamina propria, and gut-associated lymphoid tissue (62, 72, 73). Within gut-associated lymphoid tissue, lymphoid follicles mediate specific immune responses by secreting IgA (68). Damage to the intestinal immune barrier causes mucosal immune dysfunction, leading to the polarization of CD4+ T cells towards the T helper type 1 phenotype and enhancing the cytotoxicity of CD8+ T cells. This immune imbalance is characterized by the upregulation of T-box expressed in T cells, TNF-α, and interferon gamma expression, alongside increased expression of the effector T cell marker programmed cell death protein 1 (67), further exacerbating the production of pro-inflammatory cytokines. A vicious cycle ensues between immune barrier dysfunction and microbiota dysbiosis. The increase in inflammation-associated microbiota leads to the upregulation of Toll-like receptors 2/4/5 and the release of pro-inflammatory cytokines interleukin-1β and TNF-α, resulting in immune dysregulation and intestinal barrier dysfunction (67). The absence of immunomodulatory cells further exacerbates intestinal mucosal barrier damage, forming a positive feedback loop (27). As detailed in Section 3.1.3, SCFA metabolites play a crucial role in maintaining intestinal barrier integrity: butyrate inhibits histone deacetylase activity, reducing UA monosodium crystal-induced pro-inflammatory cytokine production (52); acetate mediates neutrophil caspase-dependent apoptosis through the G protein-coupled receptor 43 receptor, inhibiting inflammasome activation and promoting inflammation resolution (52). Intestinal bacteria are a significant source of purines for colon epithelial cells, and these purines are essential for maintaining intestinal barrier integrity and promoting epithelial cell proliferation. Studies have indicated that direct supplementation of purines through bacterial colonization can improve intestinal epithelial cell wound healing and barrier recovery, and adenine can also inhibit TNF-α signaling in intestinal epithelial cells, alleviating mucosal inflammation (24). Therefore, while utilizing purine-degrading bacteria to reduce pro-inflammatory UA is a potential strategy, its comprehensive impact on host intestinal health warrants thorough evaluation (24). This disruption of immune barrier function and the imbalance of repair mechanisms ultimately accelerate the progression of hyperuricemia and GA.

#### 3.2.3 Impact of the intestinal chemical barrier on hyperuricemia and gouty arthritis

The intestinal chemical barrier is composed of bile, gastric acid, and various bioactive substances such as mucopolysaccharides, glycoproteins, and glycolipids. The mucus secreted by intestinal mucosal epithelial cells contains diverse antibacterial cytokines, with mucin playing a key role in maintaining barrier function integrity by isolating epithelial cells from the intestinal lumen and scavenging reactive oxygen species (63). Damage to the chemical barrier can lead to the translocation of bacteria and viruses into the tissues surrounding the intestine, resulting in gut microbiota imbalance and metabolite disorders, which in turn affect the systemic metabolic network. In the regulation of UA metabolism, the interaction between the gut microbiota and the chemical barrier is particularly critical. Microorganisms can secrete various UA transporters, including ATP-binding cassette transporter G2, solute carrier family 2 member 9, solute carrier family 16 member 9, solute carrier family 17 members 1, 3, and 4, and solute carrier family 22 members 9, 11, and 12, which mediate UA absorption and influence fructose metabolism (25, 74, 75). Changes in fructose absorption in the intestine can lead to alterations in its luminal concentration, thereby affecting the intestinal microbial ecology. This imbalance affects the systemic metabolic network, particularly the XOD-mediated UA synthesis process. Furthermore, specific microbiota exhibit regulatory effects on the function of the chemical barrier. For instance, *Akkermansia muciniphila* participates in the systemic regulation of UA metabolism by modulating the expression of intestinal ATP-binding cassette transporter G2 and renal UA transporters such as ATP-binding cassette transporter G2, UDP-glucuronosyltransferase 1, and Glucose transporter 9 (34). Recent large-scale population-based studies have demonstrated that prior exposure to broad-spectrum antibiotics is associated with an increased risk of subsequent gout development (76), particularly in individuals receiving antibiotics with anaerobic coverage. Antibiotic-induced disruption of gut microbiota diversity and depletion of uricolytic and short-chain fatty acid–producing bacteria are believed to underlie this association. However, current evidence is primarily derived from observational studies, and a definitive causal relationship has not yet been established.

## 4 A novel perspective on gut microbiota regulation in hyperuricemia and gouty arthritis

Changes in the composition and diversity of the gut microbiota can influence the onset and progression of GA through the immune system (77). Interventions targeting the gut microbiota in GA patients aim to reshape the composition and diversity of their intestinal flora, thereby mitigating autoimmune inflammatory responses. Current research in rat models has explored the efficacy and safety of strategies such as probiotic supplementation and maintenance of intestinal barrier stability for GA (78).

### 4.1 Development of biomarkers and diagnostic tools

Recent studies have indicated that gut microbes can serve as non-invasive diagnostic markers for various diseases (79). In the pathogenesis of hyperuricemia and GA, specific gut microbiota and their metabolites participate in the disease process by modulating UA metabolism, inflammatory immune responses, and intestinal barrier function. Compared to traditional serum UA testing, detection methods based on the gut microbiota exhibit higher sensitivity and are non-invasive. Research has revealed significant characteristic alterations in the gut microbiota of GA patients: a decrease in *Phascolarctobacterium faecium* and butyrate-producing bacteria, while the levels of *Mucispirillum schaedleri* and *Xylanibacter* are elevated, and *Faecalibacterium prausnitzii* and *Pseudobutyrivibrio xylanivorans* are significantly reduced (16). Based on these characteristics, researchers constructed a diagnostic model incorporating 17 GA-related bacteria, achieving an accuracy of 88.9% in a validation cohort of 15 subjects, outperforming serum UA detection methods (57). Among these, butyrate, a major metabolite produced by the gut microbiota, plays a crucial role in regulating inflammatory balance and is an important microbial metabolic marker significantly associated with hyperuricemia and GA (42).

### 4.2 Gut microbiota-based therapies for hyperuricemia and gouty arthritis

**Figure 3:**
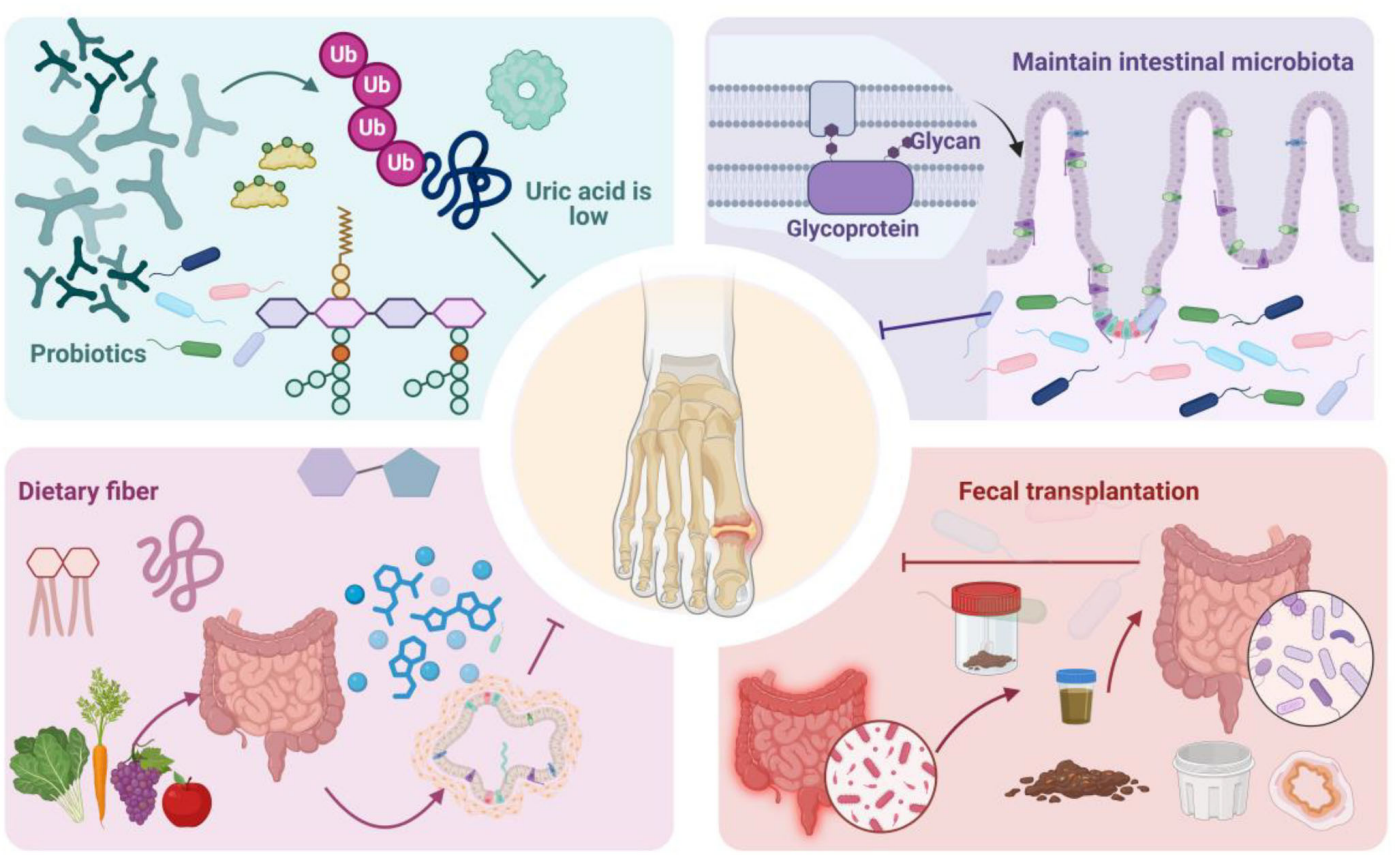
schematizes a gut micro-ecosystem-based therapeutic strategy for hyperuricemia and GA. Gut microbiome modulation has emerged as a promising therapeutic avenue. Therapeutic modalities encompassing probiotic administration, FMT, dietary fiber supplementation, and maintenance of intestinal barrier integrity are efficacious in impeding GA progression. Modulating the intestinal micro-ecosystem can effectively reduce uric acid concentrations, mitigate the incidence of inflammation, and restore intestinal micro-ecological equilibrium, thus providing novel therapeutic targets for GA management.

Gut microbiota modulation has emerged as a novel therapeutic strategy (33). Studies have shown that regulating the gut microbiota can influence multiple key therapeutic targets. **Figure 3** illustrates a schematic diagram of gut microbiota-based therapies for hyperuricemia and GA. Microbiota modulation can reverse UA metabolism in the gut and enhance UA excretion. Furthermore, alterations in the gut microbiota can affect serum UA levels by modulating host metabolites. With increasing understanding of the role of the gut microbiota in the pathogenesis of hyperuricemia and GA, biological therapies such as probiotics and fecal microbiota transplantation (FMT) have garnered significant attention. These therapeutic approaches primarily function by inhibiting purine metabolism and inflammatory factors, regulating the expression of transporter proteins, and protecting intestinal barrier integrity. Simultaneously, they can increase the abundance of SCFA-producing bacteria, promoting serum and hepatic NADPH oxidase activity (80).

#### 4.2.1 Probiotic therapy

Probiotic research has indicated that *Bifidobacterium* and *Lactobacillus* are the primary functional strains, with newly discovered *Pseudobutyrivibrio xylanivorans*, *Akkermansia muciniphila*, and *Clostridium* species also demonstrating significant therapeutic effects (81). In clinical studies, a comparison of 20 GA patients and 20 healthy controls revealed lower microbial diversity in GA patients, where alterations in *Prevotella copri*, *Bacteroides uniformis*, and *Erysipelotrichaceae* led to abnormalities in UA and purine metabolism pathways, subsequently affecting the levels of metabolites such as bile salts and cholesterol (82). These differentially abundant species showed a significant correlation with UA concentration, suggesting their potential as therapeutic targets (82). In a randomized, double-blind clinical trial involving 120 volunteers, supplementation with probiotic yogurt containing *Lactobacillus fermentum* GR-3 significantly reduced UA levels and inflammatory markers in high-risk individuals (83). Metabolomic analysis revealed that regulating gut microbiota such as *Prevotella copri* and *Bifidobacterium animalis* subsp. *lactis* can lower UA levels in patients with hyperuricemia and GA. Animal experiments have further confirmed the therapeutic effects of probiotics: *Lactobacillus fermentum* JL-3 was able to alleviate gut microbiota dysbiosis induced by hyperuricemia and reduce UA levels in a mouse model (84); the probiotic strain *DM9218* improved fructose-induced hyperuricemia by reducing serum UA levels and hepatic XOD activity (85); and a uricase-producing strain reduced serum UA levels and improved hypertension and kidney disease in a hyperuricemic animal model (86). *Lactobacillus* probiotics have shown significant efficacy. *Lactobacillus reuteri* and *Lactobacillus fermentum* reduce UA levels by inhibiting purine synthesis, while *Lactobacillus brevis* DM9218 and *Lactobacillus gasseri* PA3 can effectively degrade purine metabolism intermediates (87, 88). Despite the promising outlook for probiotic therapy, its protective mechanisms have not been fully elucidated, and the efficacy of low-dose usage, in particular, warrants further investigation.

Despite promising preclinical and early clinical results, several challenges limit the widespread clinical application of probiotics for gout. First, strain-specific efficacy remains a major concern. Not all Lactobacillus or Bifidobacterium strains exhibit uricolytic activity or anti-inflammatory effects. Current evidence derives primarily from specific strains (e.g., Lactobacillus fermentum GR-3, Lactobacillus brevis DM9218, Lactobacillus gasseri PA-3), and results cannot be generalized across the genus. Comprehensive strain characterization and mechanism elucidation are required before clinical recommendations can be standardized. Second, limited bioavailability and colonization present significant obstacles. Many probiotic strains demonstrate poor survival through gastric acid (pH 1.5-3.5) and bile salts (0.3-0.5% concentration in duodenum), resulting in insufficient colonization density to exert therapeutic effects. Encapsulation technologies and targeted delivery systems are being developed but not yet widely implemented. Third, individual variation in response creates therapeutic unpredictability. Gut microbiota composition varies significantly among individuals based on genetics, diet, age, medication history, and baseline microbiome profile. Clinical trials report response rates of 40-60%, with substantial non-responder populations. Personalized approaches based on baseline microbiota profiling may improve efficacy but require further validation. Fourth, insufficient long-term safety data limits confident clinical recommendations. Most clinical studies report outcomes at 12 weeks or less. Long-term effects (greater than 6 months) on gut microbiome stability, immune function, and potential adverse events remain unclear. Concerns about probiotic translocation in immunocompromised patients and potential for antibiotic resistance gene transfer warrant careful monitoring. Fifth, dose-response uncertainties complicate treatment protocols. Optimal dosing regimens (CFU count, frequency, duration) have not been systematically established. Current recommendations range from 10^8 to 10^11 CFU/day, based primarily on manufacturing feasibility rather than pharmacokinetic/pharmacodynamic studies. Dose-response trials are needed to establish evidence-based guidelines. Sixth, lack of standardization and quality control undermines therapeutic reliability. Commercial probiotic products often contain viable counts lower than labeled claims, inconsistent strain composition, and potential contamination. Regulatory oversight varies by country, and many products marketed for gout lack clinical validation. Stricter quality control standards and third-party verification are essential. To address these limitations, future research should prioritize mechanistic studies elucidating strain-specific effects, development of next-generation probiotics with enhanced survival and colonization, large-scale randomized controlled trials with long-term follow-up (1 year or longer), personalized treatment algorithms based on microbiota signatures, and establishment of regulatory frameworks for probiotic therapeutics.

#### 4.2.2 Targeting the gut microbiome

Plant polysaccharides optimize the gut microenvironment by improving intestinal tissue morphology, maintaining barrier integrity, enhancing immune responses, and modulating gut microbiota composition (89). As a carbon source for specific gut microorganisms, polysaccharides may be key components in regulating microbiome activity during fermentation. Among these, seaweed polysaccharides have shown significant UA-lowering effects: *Ulva pertusa* polysaccharide regulates the gut microbiota by increasing the abundance of beneficial bacteria and reducing the proportion of harmful bacteria, thereby lowering UA levels (90). Polysaccharides derived from *Enteromorpha prolifera* can significantly reduce serum UA, blood urea nitrogen, and serum and hepatic XOD levels in hyperuricemic mice. Research has confirmed that polysaccharides from *Enteromorpha prolifera* maintain gut microbiota homeostasis by increasing the relative abundance of *Alistipes* and *Parasutterella*, whose abundance is negatively correlated with UA levels (91).

#### 4.2.3 Dietary fiber

Dietary fiber, an essential nutrient for the human body, plays a crucial role in immune and metabolic regulation by modulating the structure of the gut microbiota (92, 93). Supplementation with dietary fiber can significantly alter the diversity of the gut microbiota and increase the abundance of SCFA-producing bacteria (94, 95). Among these, inulin alleviates hyperuricemia by upregulating intestinal *ATP-binding cassette transporter G2* expression and downregulating hepatic XOD expression and activity (96). Studies have shown that inulin can increase the relative abundance of SCFA-producing bacteria such as *Akkermansia* and *Ruminococcus*, and increase the production of acetate, propionate, and butyrate, which is positively correlated with its UA-lowering effect (96). Furthermore, supplementation with β-carotene and green tea powder rich in dietary fiber can reduce serum UA and pro-inflammatory factors interleukin-1β, interleukin-6, and TNF-α levels in a GA mouse model by regulating the gut microbiota and inhibiting purine and pyrimidine metabolism (97).

#### 4.2.4 Fecal microbiota transplantation

FMT, a therapeutic approach involving the transplantation of gut microbiota from a healthy donor into a recipient via the digestive tract to restore intestinal microbial diversity, has become an important direction in clinical research. Given the close association between serum UA concentration and gut microecological balance, FMT offers a novel strategy for the treatment of hyperuricemia and GA. Clinical studies have shown that FMT can reduce serum UA levels in GA patients and decrease the frequency and duration of acute GA attacks (33). Simultaneously, FMT can lower diamine oxidase and endotoxin levels, improving impaired intestinal barrier function (98). Animal experiments have further confirmed the potential value of FMT in the treatment of hyperuricemia: transplantation of fecal microbiota from mice treated with *Gastrodia elata* polysaccharide or carrageenan oligosaccharide can transfer their UA-lowering effects (99, 100). Furthermore, transplantation of fecal microbiota from goose gizzard-processed mice also demonstrated significant anti-hyperuricemic effects, with the mechanism involving the restoration of gut microbiota balance, repair of the intestinal epithelial barrier, and promotion of SCFA production (101). Although the long-term effects of FMT on human health require further clinical evidence, it offers a new option for the treatment of hyperuricemia and GA with significant potential for clinical application.

## 5 Conclusions

This review demonstrates that gut microbiota dysbiosis plays a pivotal role in hyperuricemia and gout pathogenesis through three interconnected mechanisms: dysregulated uric acid metabolism via altered microbial enzymes and transporter expression, disrupted intestinal barrier integrity across physical, immune, and chemical barriers, and aberrant immune activation characterized by TLR4/NF-κB priming, NLRP3 inflammasome assembly, and IL-1β-mediated inflammatory cascades. Microbiota-targeted interventions—including probiotics, prebiotics, and fecal microbiota transplantation—show therapeutic promise, but significant knowledge gaps remain. Several key unresolved questions demand attention. Which specific microbial strains or gene clusters exhibit causal (not merely correlative) relationships with gout? Can gut microbiota signatures predict gout flare risk or treatment response to urate-lowering therapy? What are the optimal timing, duration, formulation, and dosing strategies for probiotic interventions? How do host genetics (e.g., ABCG2 polymorphisms, HLA-B*5801) interact with microbiota composition to modify gout susceptibility and severity? Do prior antibiotic exposures create long-lasting alterations in purine-degrading bacterial communities? Future research priorities should focus on five critical areas. First, multi-omics integration combining metagenomics, metabolomics, and immunophenotyping in longitudinal cohorts will map comprehensive mechanistic pathways. Second, causality establishment employing germ-free animal models, human FMT studies, and defined bacterial consortia will distinguish causative from consequential microbiota changes. Third, precision medicine development creating microbiota-based stratification systems will enable personalized treatment selection. Fourth, large-scale validation through adequately powered randomized controlled trials with follow-up of 1 year or longer will establish clinical efficacy and safety. Fifth, mechanistic target identification defining druggable targets within microbial metabolic pathways (e.g., specific enzymes in purine catabolism) will guide next-generation therapeutics. The convergence of microbiome science, immunology, and metabolomics has revealed the gut-joint axis as a critical regulator of gout pathogenesis. Translating these mechanistic insights into clinically effective, microbiota-targeted therapies requires moving beyond descriptive studies toward intervention-based research that can deliver tangible benefits to gout patients.
